# Supplementary material for: Polyindole-Derived Nitrogen-Doped Graphene Quantum Dots-Based Electrochemical Sensor for Dopamine Detection
Source: Biosensors (Basel). 2022 Nov 22;12(12):1063. doi: 10.3390/bios12121063 (PMC9775058; doi:10.3390/bios12121063)
Supplement: Supplementary file 1 [file biosensors-12-01063-s001.zip › biosensors-2017573-supplementary.pdf]

biosensors

Supporting Information

Polyindole-Derived Nitrogen-Doped Graphene Quantum Dots-Based Electrochemical Sensor for Dopamine Detection

Anjitha Thadathil [1], Dipin Thacharakkal [1], Yahya A. Ismail [1] and Pradeepan Periyat [2,*]

[1] Department of Chemistry, University of Calicut, Malappuram 673635, India
[2] Department of Environmental Studies, Kannur University, Kannur 670567, India
* Correspondence: pperiyat@kannuruniv.ac.in

Supporting information Scheme S1

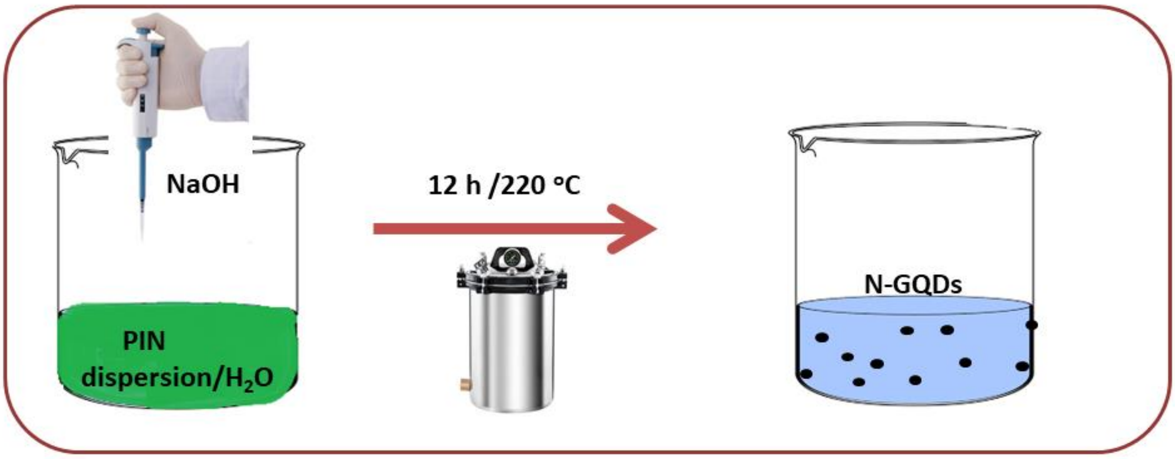

Scheme S1. Schematic of the synthesis of N-GQDs.

Supporting information Scheme S2

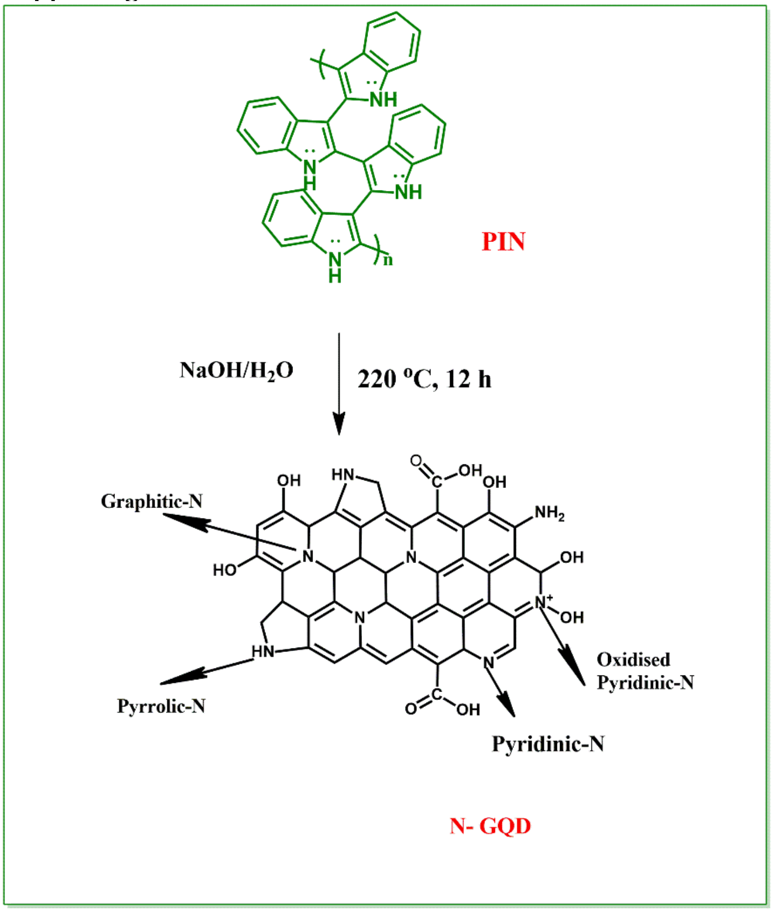

Scheme S2. The proposed structure of N-GQDs synthesized from

PIN. **Supporting information Figure S1**

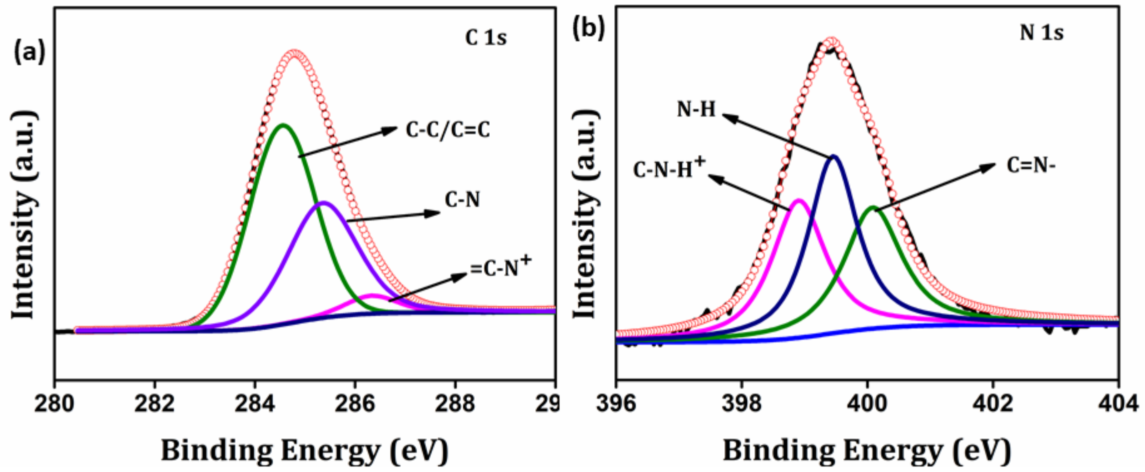

Figure S1. (**a**,**b**)High-resolution XPS spectra of the C 1s, N 1s, and O 1s of PIN, respectively, and their related curve-fitted components.

Supporting information Figure S2

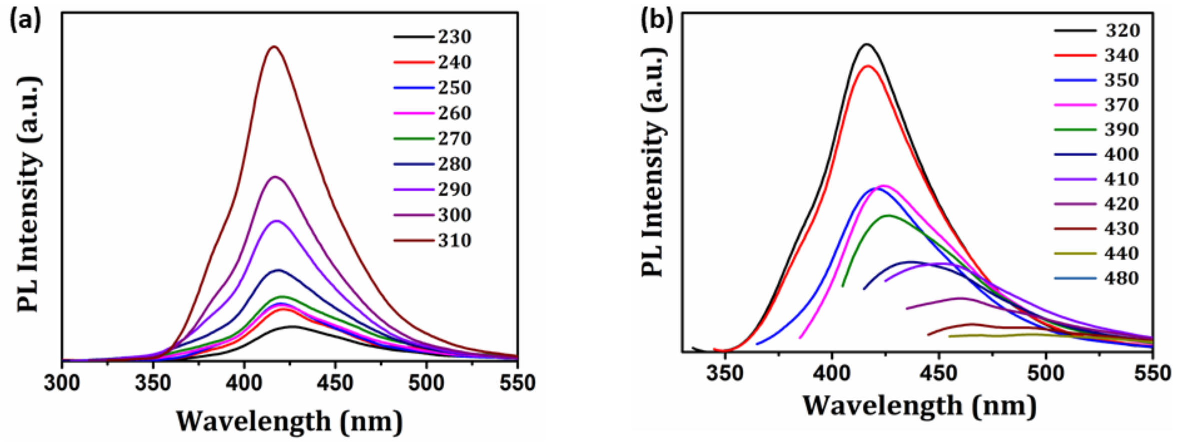

Figure S2. (**a**,**b**) Dependence of the PL spectra of N-GQDs on the excitation wavelength from 230 to 480 nm.

On excitation with 230 nm, the PL spectrum of N-GQDs exhibited an emission peak at ~425 nm with a Stokes shift of 195 nm, corresponding to a blue emission; with longer excitation wavelengths (from 230 to 310 nm), the PL peak slightly shifted to lower wavelengths with a rapid increase in intensity, and the fluorescence intensity reached the maximum value when the excitation wavelength was set at 310 nm (Figure S2a). From an excitation wavelength of 320 nm onward, the strong emission band at 416 nm showed a trend of shift to a longer wavelength, and intensity decreased with longer excitation wavelengths (320–480 nm) (Figure S2b). Although the exact mechanisms responsible for N-GQDs photoluminescence remain to be elucidated, it is supposed that the luminescence relates to the sp^3 matrix. Previous studies report that the isolated sp^2-hybridized clusters with a size of ca. 3 nm within the carbon–oxygen matrix can yield band gaps consistent with blue emission owing to the localization of electron–hole pairs.

Supporting information Figure S3

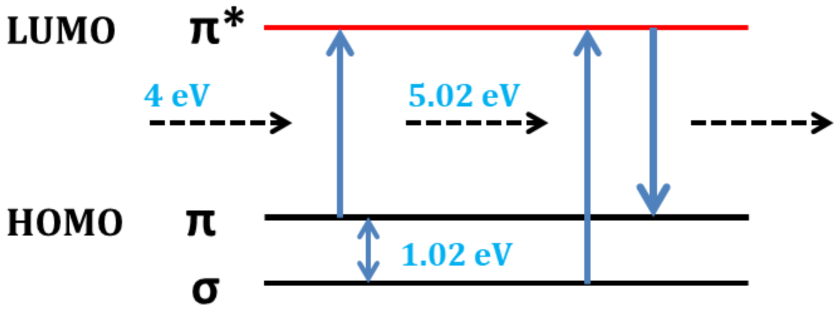

Figure S3. A diagram of the electronic transition of triple carbenes at zigzag sites observed in the optical spectra of the N-GQDs.

Dengyu Pan et al. [99] proposed that the luminescence in GQDs may have originated from free zigzag sites with a carbene-like triplet ground state. The GQDs show strong luminescence, most likely because there is a high concentration of free zigzag sites owing to the small diameter (ca. 4.2 nm). For the triplet state of carbene ($\sigma^1\pi^1$), the two orbitals σ and π are singly occupied. Based on Hoffmann's calculations [100], the energy difference (dE) between the s and p orbitals for a triplet ground state should be below 1.5 eV. Since the triple carbenes are most common at zigzag edges, the peak at 247 nm (5.02 eV) and

310 nm (4 eV) observed in the PLE spectra of N-GQDs can be attributed as transitions from the σ and π orbitals (highest occupied molecular orbitals, HOMOs) to the lowest unoccupied molecular orbital (LUMO), as illustrated in Figure S3. Thus, dE is determined to be 1.02 eV, which is within the required value (<1.5 eV) for triple carbenes and proposes that the assignment of the two transitions is reasonable. Since the two transitions are closely connected to the emitted PL of N-GQDs, the detected cyan blue PL emission is attributed to the irradiation decay of the activated electrons from LUMO to HOMO.

References

99 Pan, D; Zhang, J; Li, Z; Wu, M. Hydrothermal route for cutting graphene sheets into blue-luminescent graphene quantum dots. *Adv. Mater.* **2010**, *22*, 734–738.

100 Hoffmann, R. Trimethylene and the addition of methylene to ethylene. *J. Am. Chem. Soc.* **1968**, *90*, 1475–1485.

Supporting information Figure S4

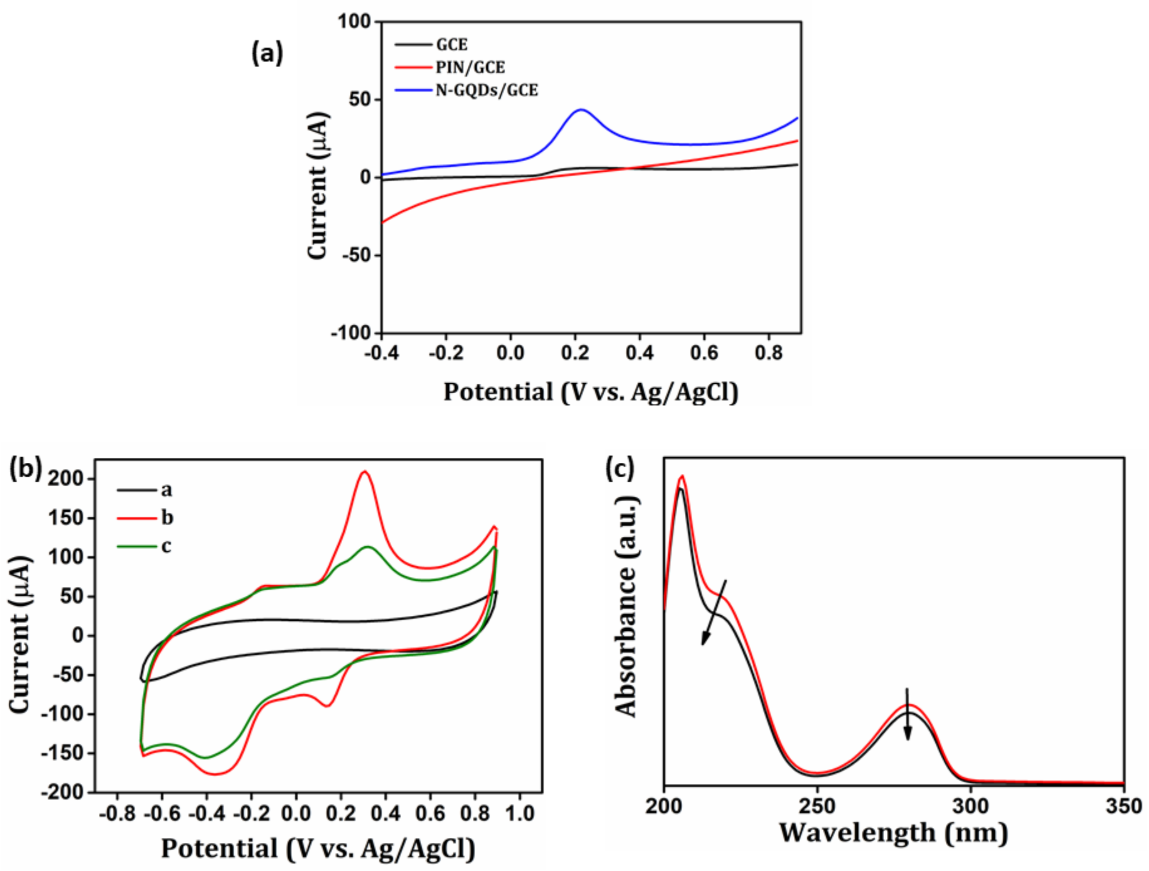

Figure S4. (**a**)LSV of GCE, PIN/GCE, and N-GQDs/GCE in PBS (0.1 M, pH 7.4). (**b**) CVs of N-GQDs/GCE in PBS (0.1 M, pH 7.4) without DA (trace a). After that, electrodes were immersed in 1 mM of DA for 5 min (trace b) and after washing with distilled water (trace c) (scan rate: 50 mV/s). (**c**) UV-Vis spectra of 1 mM of DA before (curve red) and after being accumulated by N-GQDs/GCE (curve black).

The higher electrocatalytic activity of the N-GQDs/GCE (Figure S4a) in comparison with PIN/GCE and GCE can be ascribed to the improved electronic characteristics. The higher activity of the N-GQDs/GCE can also be ascribed to their enhanced capability to adsorb DA. The adsorption is important because it is an essential first step for DA reduction to occur. To demonstrate the adsorption ability of N-GQDs/GCE, we performed a pre-concentration experiment (Figure S4b). First, the N-GQDs/GCE was immersed in 0.1 M PBS, and the electrochemical response was evaluated. It is clear from the observed CV

response that in the absence of DA, no significant CV peaks are observed. Further, the N-GQDs/GCE was immersed in 1 mM DA solutions (0.1 M of PBS) for 5 min. Then, the electrode was taken out and washed with distilled water, and then kept in PBS solution (without DA) to record the CV response. The obtained CV response is depicted in Figure S4b, implying that the adsorption of DA on N-GQDs/GCE is stable. Furthermore, we monitored the adsorption of DA on N-GQDs/GCE by UV-Vis spectroscopy. The UV-Vis absorption of the DA solution (0.1M of PBS) before and after CV measurements on the N-GQDs/GCE was taken, and the respective spectra are shown in Figure S4c. It is obvious that the intensities of the UV-Vis peak of DA decreased after adsorption. The intensities decreased by 10.2% after DA adsorption, suggesting a higher tendency for DA adsorption on N-GQDs.

Supporting information Figure S5

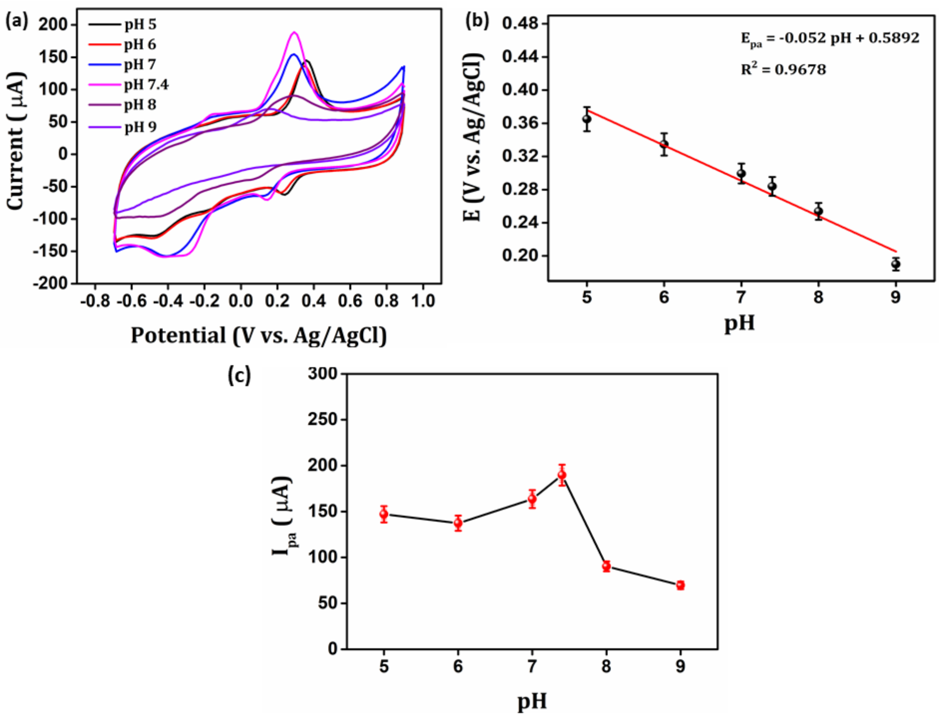

Figure S5. (**a**) CVs of 1 mM of DA (0.1 M PBS) with different pH values (5–9) at N-GQDs/GCE. (**b**) Linear relation between the potential (E) of DA (mV) and the pH value. (**c**) Relation between I_{pa} and pH values (scan rate, 50 mV s^{-1}).

With the pH increment, the E_{pa} for DA shifted cathodically, indicating that the number of electrons and protons is the same throughout the electrochemical oxidation process of DA. The pH introduced a line with the equation: E_{pa} (V) = -0.052pH + 0.5892 (R^2 = -0.9678). As shown in Figure S5b, the DA anodic peak potential is linearly proportional to the solution pH with a slope of -0.052 V per unit pH; this is very near to the theoretical Nernstian slope (-0.059 V), which supports the $2e^-/2H^+$ redox reaction system, since two electrons are transferred. Further, Figure S5c displays that the anodic peak currents (I_{pa}) increase steadily with the pH varying from 5.0 to 7.4, then drop sharply as the pH reaches 9. This phenomenon could be ascribed to the charged state balance between dopamine and the residual carboxyl and hydroxyl groups of N-GQDs.

Supporting information Figure S6

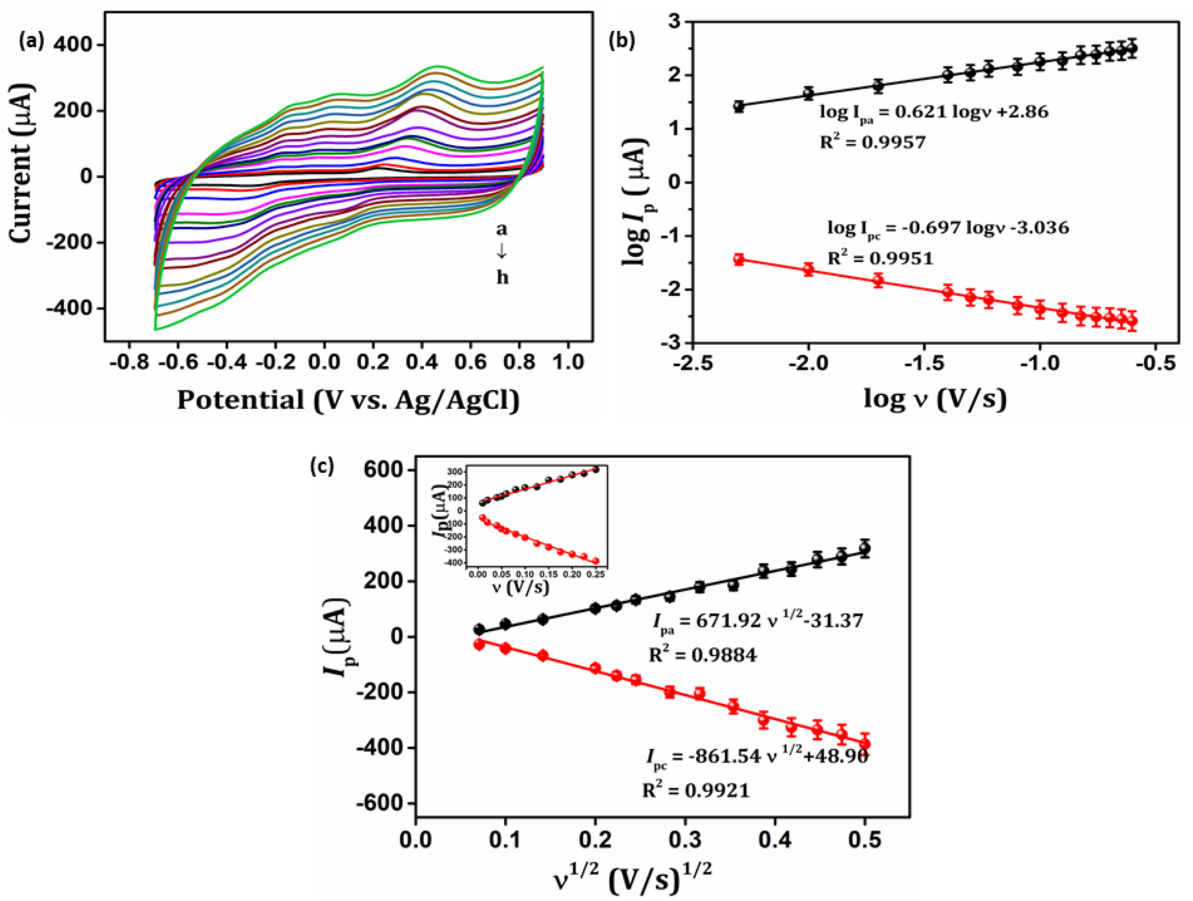

Figure S6. (**a**) CV of DA (1 mM) on N-GQDs/GCE with different scan rates (a-h: 0.010, 0.025, 0.050, 0.075, 0.10, 0.15, 0.20, and 0.25 V s^{-1}, respectively) (pH = 7.4); (**b**) log I_p versus log ν; (**c**) I_{pa} and I_{pc} versus $v^{1/2}$.

The heterogeneous electron transfer rate (K_s) in the redox probe can be determined using the equation,

$$K_s = \frac{\Delta E_p n F \vartheta}{RT} \quad (1)$$

where ΔE_p (separation potential) = 0.158 V and v = 50 mV /s. The estimated value of k_s is 3.01 s^{-1}, which is significantly higher than that reported earlier [71,89,101,102].

References

71 Thomas, D.; Rasheed, Z.; Jagan, J.S.; Kumar, K.G. Study of kinetic parameters and development of a voltammetric sensor for the determination of butylated hydroxyanisole (BHA) in oil samples. *J. Food Sci. Technol.* **2015**, *52*, 6719–6726.

89 Huang, Q.; Lin, X.; Tong, L.; Tong, Q.-X. Graphene quantum dots/multiwalled carbon nanotubes composite-based electrochemical sensor for detecting dopamine release from living cells. *ACS Sustain. Chem. Eng.* **2020**, *8*, 1644–1650.

101 Zhang, L.; Jiang, X.; Wang, E.; Dong, S. Attachment of gold nanoparticles to glassy carbon electrode and its application for the direct electrochemistry and electrocatalytic behavior of hemoglobin. Biosensors and Bioelectronics **2005**, *21*, 337–345.

102 Topoglidis, E.; Astuti, Y.; Duriaux, F.; Grätzel, M.; Durrant, J.R. Direct electrochemistry and nitric oxide interaction of heme proteins adsorbed on nanocrystalline tin oxide electrodes. *Langmuir* **2003**, *19*, 6894–6900.

Supporting information Figure S7

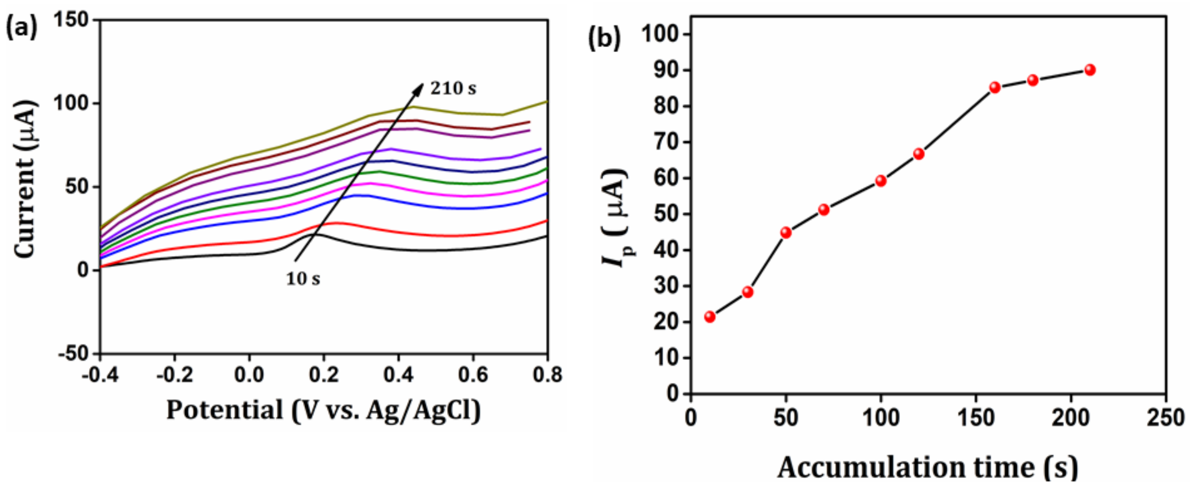

Figure S7. (**a**) LSV responses with accumulation time. (**b**) Dependence of I_P on accumulation time.

Supporting information Figure S8

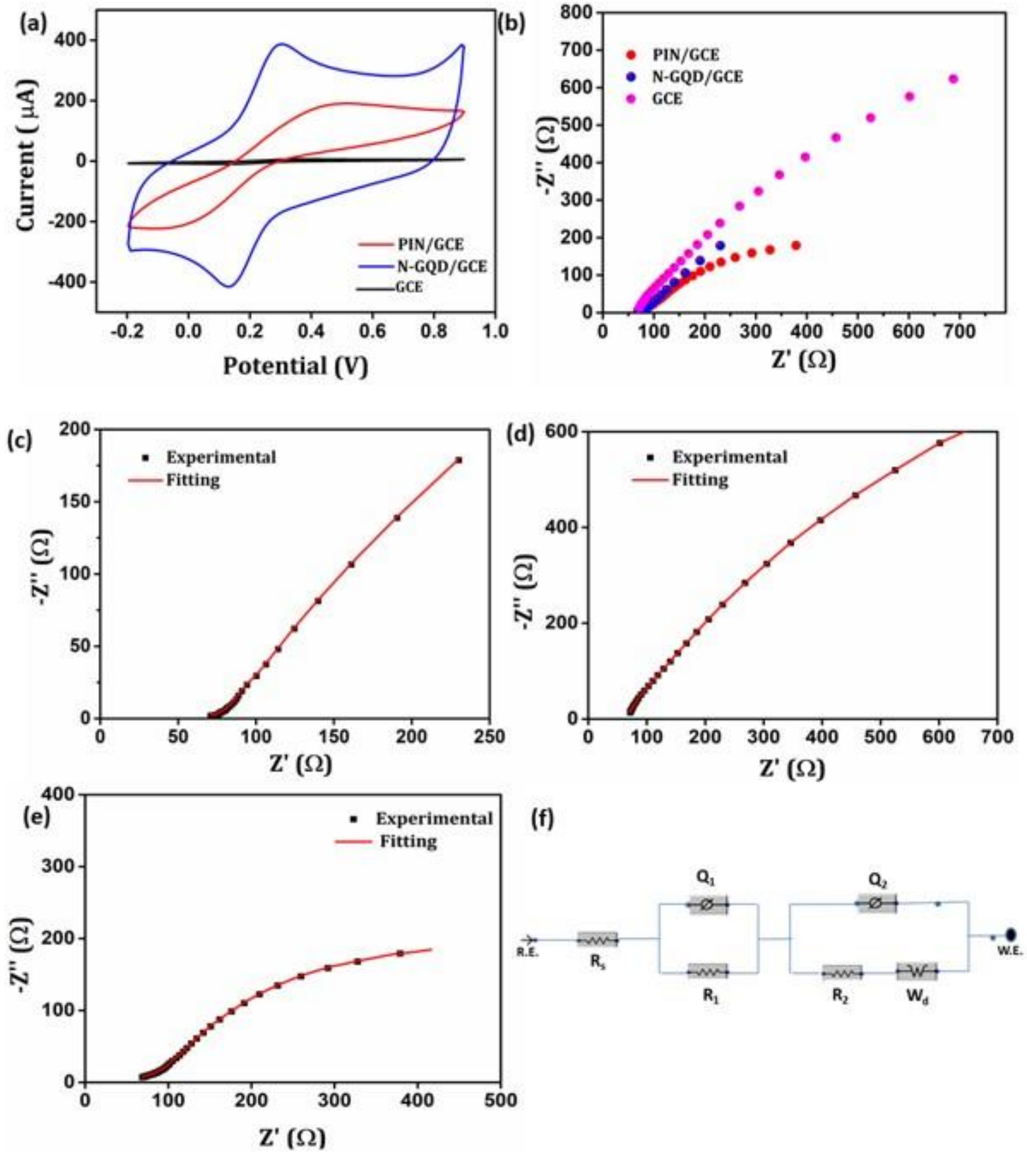

Figure S8. (**a**) Cyclic voltammograms obtained at PIN/GCE and N-GQDs/GCE containing 1 mM of $K_3[Fe(CN)_6]$ in 0.1 M KCl at a scan rate of 100 mV s^{-1}; (**b**) Nyquist plots of GCE, PIN/GCE, and N-GQDs/GCE in 0.1 M of KCl solution containing 5 mM of $K_3Fe(CN)_6$/$K_4Fe(CN)_6$ (1:1) in the frequency range from 0.1 Hz to 100 kHz. (**c**,**d**,**e**) Nyquist plots of GCE, PIN/GCE, and N-GQDs/GCE, respectively after fitting with an equivalent circuit. (**f**) Equivalent circuit used for fitting the Nyquist plots.

To evaluate the electrochemical performance, the CVs of different electrodes were recorded in the mixture solution of 1.0 mM of $Fe(CN)_6^{3-}$ and 0.1 M of KCl (Figure S8a). A pair of reversible redox peaks are observed on the PIN/GCE, with the anodic peak current (I_{pa}) of 190.36 μA and cathodic peak current (I_{pc}) of 220.45μA, respectively. Here, the potential separation (E_p) between the anodic peak and the cathodic peak is 53 mV. However,

$Fe(CN)_6^{3-}$ oxidation and reduction have higher peak currents (I_{pa} = 389.68 μA and I_{pc} = -417.13 μA) and smaller peak potential separation (ΔE_p, ~16 versus 53 mV) on the N-GQD/GCE compared to the PIN/GCE. This specifies that the N-GQDs/GCE has greater electrocatalytic activity and is more reversible than the PIN/GCE for the model reaction, $Fe(CN)_6^{3-}$ electro-oxidation, and reduction. Furthermore, their electrochemical active areas were calculated as 0.003 cm^2, 0.115 cm^2, and 0.283 cm^2 for GCE, PIN/GCE, and N-GQDs/GCE, respectively, using the Randles–Sevcik equation, while the geometric area calculated for GCE was 0.0088 cm^2.

Figure S8b shows the Nyquist plots of GCE, PIN/GCE, and N-GQDs/GCE in 0.1 M of KCl solution containing 5 mM of $K_3Fe(CN)_6/K_4Fe(CN)_6$ (1:1) in the frequency range from 0.1 Hz to 100 kHz. The minimum diffusion resistance of N-GQD/GCE is validated from the steepest slope of the straight line in the lower frequency area. The minimum diffusion resistance of N-GQDs/GCE is validated from the steepest slope of the straight line in the lower frequency area. The interfacial charge transfer resistance (R_{ct}) of N-GQDs/GCE is much smaller than the PIN/GCE, which suggests that N-GQDs/GCE can construct good electron-transferred pathways between the electrode and electrolyte.

Supporting information Table S1

Table S1. Fitted values of the components R_s, R_1, R_2, and W_d in equivalent circuit for different electrodes.

**Electrode**	**R_s (Ω)**	**R_1 (Ω)**	**R_2 (Ω)**	**W_d**
GCE	89	14	134	200
PIN/GCE	75	31	0.6	213
N-GQDs/GCE	70	7.6	0.419	19

Supporting information Figure S9

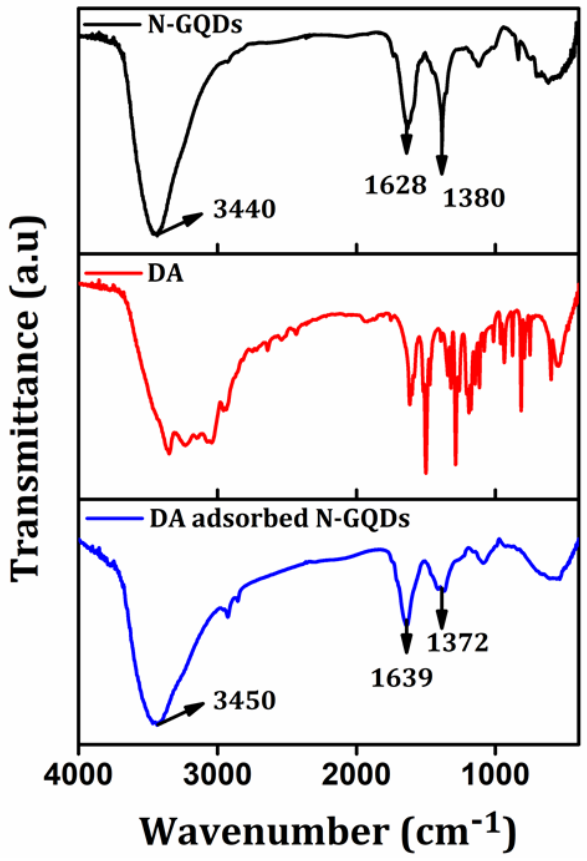

Figure S9. The FT-IR spectra of N-GQDs, DA, and DA-adsorbed N-GQDs.

Supporting information Figure S10

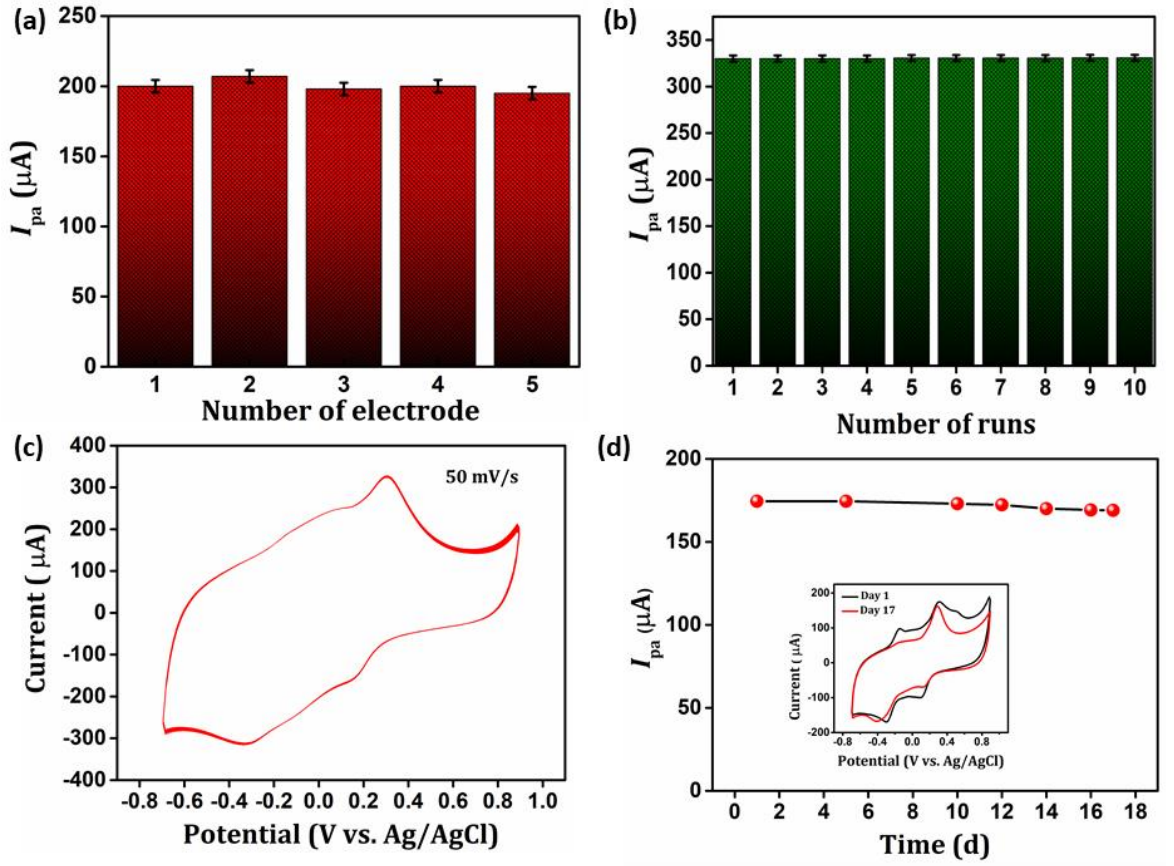

Figure S10. (**a**) Reproducibility of N-GQDs/GCE for five different modified electrodes. (**b**) Repetitive measurement of the N-GQDs/GCE with single modified electrodes. (**c**) Cyclic voltammograms (50 cycles) confirm the stability of the sensor. (**d**) Stability investigation by recording the anodic peak currents for 17 days.

Supporting information Table S2

Table S2. Evaluation of the accuracy and precision of the proposed method for determination of DA in real urine samples.

Samples	Concentration of DA Added (μM)	Concentration of DA Found (μM)	Recovery (%)	Error (%)
1	0	0	-	-
2	5	4.92	98.4	1.6
3	10	9.87	98.7	1.3
4	15	14.88	99.2	0.8
5	20	19.65	98.25	1.75
6	25	25.74	102.29	2.96

Supporting information Figure S11

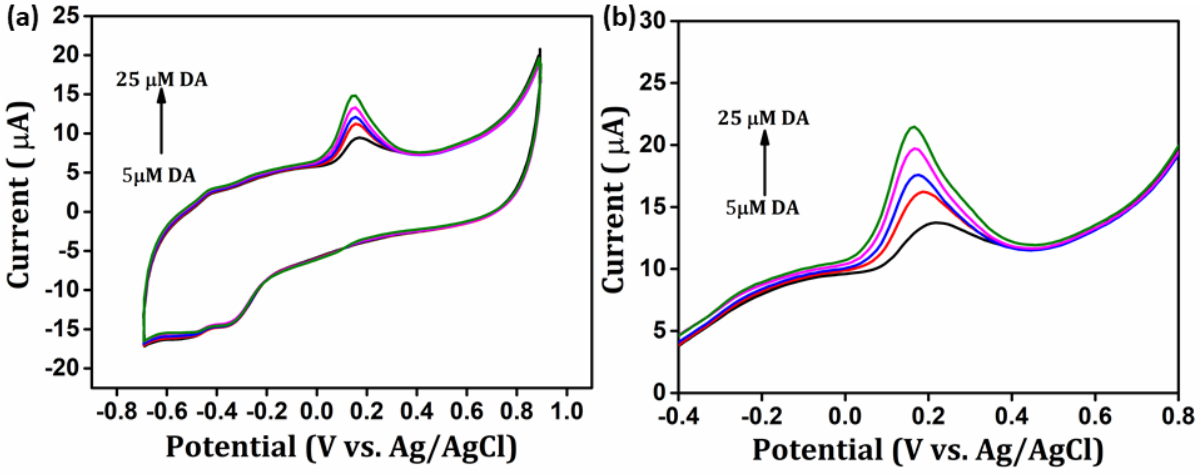

Figure S11. (a) CVs of DA in urine at N-GQDs/GCE for concentrations from 5 to 25 μM. (b) LSVs of DA in urine at N-GQDs/GCE for concentrations from 5 to 25 μM.
